# Cell–gel interactions of in-gel propagating bacteria

**DOI:** 10.1186/s13104-018-3811-x

**Published:** 2018-10-04

**Authors:** Philip Serwer, Barbara Hunter, Elena T. Wright

**Affiliations:** 1 Department of Biochemistry and Structural Biology, The University of Texas Health Science Center, 7703 Floyd Curl Drive, San Antonio, TX 78229-3900 USA; 2 Department of Pathology, The University of Texas Health Science Center, 7703 Floyd Curl Drive, San Antonio, TX 78229-3900 USA

**Keywords:** Bacterial clustering, Bacterial plasticity, Electron microscopy, In-gel bacterial propagation, Thin sections

## Abstract

**Objective:**

Our immediate objective is to test the data-suggested possibility that in-agarose gel bacterial propagation causes gel fiber dislocation and alteration of cell distribution. We also test the further effect of lowering water activity. We perform these tests with both Gram-negative and Gram-positive bacteria. Data are obtained via electron microscopy of thin sections, which provides the first images of both bacteria and gel fibers in gel-supported bacterial lawns. The long-term objective is analysis of the effects of in-gel propagation on the DNA packaging of phages.

**Results:**

We find that agarose gel-supported cells in lawns of *Escherichia coli* and *Lysinibacillus* (1) are primarily in clusters that increase in size with time and are surrounded by gel fibers, and (2) sometimes undergo gel-induced, post-duplication rotation and translation. Bacterial growth-induced dislocation of gel fibers is observed. One reason for clustering is that clustering promotes growth by increasing the growth-derived force applied to the gel fibers. Reactive force exerted by gel on cells explains cell movement. Finally, addition to growth medium of 0.94 M sucrose causes cluster-associated *E. coli* cells to become more densely packed and polymorphic. Shape is determined, in part, by neighboring cells, a novel observation to our knowledge.

## Introduction

Bacterial cells propagate in a polymer gel when in either (1) a lawn for plaque assay of phages (recent references [1, 2]) or (2) a biofilm [3–5]. But, the supporting gel’s influence on the cells is not known. Agar gels are typically used [1, 2], although recent studies have shown that polyacrylamide gels can also be used [6]. Analysis is optimally done with procedure, such as used here, that reveals both cells and gel fibers.

In theory, bacterial propagation in an agarose gel is constrained by agarose fibers, which are rigid to thermal motion [7]. Indeed, if these fibers are unbroken and rigid to bacterial motion, in-gel bacteria could not duplicate when the diameter of the gel’s effective pore (2 × *P*_E_) is smaller than bacterial cell dimensions. In liquid media, *Escherichia coli* has diameter of ~ 1000 nm and length of 2000–5000 nm [8]. Studies of gel electrophoretic sieving yield a smaller 2 × *P*_E_ of 462 nm for 0.6% underivatized agarose; *P*_E_ decreases as agarose concentration increases [9]. But, here we propagate *E. coli* in 0.6% agarose gels. Also, elongation of single *E. coli* cells is observed by light microscopy in (1) 1% hydroxyethyl agarose gels [10] (*P*_E_ = 113 nm [9]) and (2) 8% underivatized agarose gels [11] (2 × *P*_E_ < 60 nm [9]). Tryptone, as used in media here, combines with bacteria to increase gel stiffness [10].

In-gel propagability of *E. coli* suggests, therefore, that cell growth-induced dislocation of gel fibers occurs. However, no observation has, to our knowledge, been made of gel fibers after in-gel bacterial duplication. Our interest originated during analysis of in-plaque phage DNA packaging. Here, we use electron microscopy (EM) of thin sections to achieve this observation for bacterial propagation in agarose gels; gel swelling during sectioning prevents this analysis of polyacrylamide gels [12].

## Main text

### Methods

#### Bacterial strains

*Escherichia coli* BB/1 (host for phages T3 and T7 [13]) and a *Lysinibacillus* are used here as Gram-negative and Gram-positive bacteria, respectively. The *Lysinibacillus* is host for phage G (*Lysinibacillus* PGH). It was typed via commercial sequencing and informatic analysis (J.A. Thomas, W. Jiang and P. Serwer, unpublished). It had previously been mis-identified as *Bacillus megaterium* PGH [14], which, in contrast to observations made here, has diameter 1.5× larger than that of *E. coli* [15].

*Escherichia coli* BB/1 was propagated in 2 × LB medium: 20 g tryptone, 10 g yeast extract, 5 g NaCl in 1.0 l of Milli-Q filtered water (Millipore/Sigma). A liquid culture was propagated to stationary phase at 30 °C just before use. The *Lysinibacillus* was thusly propagated in medium with 5 g KCl, 10 g Bacto tryptone per ml of water with sterile 0.001 M CaCl_2_ subsequently added.

#### In-gel propagation

Gels for in-gel propagation were prepared by autoclaving and dissolving solid Seakem Gold (Lonza) agarose in a medium specified above. This solution was equilibrated at 50 °C. Then, 3 drops (*Lysinibacillus*) or 5 drops (*E. coli*) of bacterial culture were mixed with 3.5 ml of the molten agarose. This mixture was poured into a Petri plate over a 1.0% bottom layer agar gel in 10 g Bacto tryptone, 5 g NaCl in 1.0 l of water, with sterile 0.001 M CaCl_2_ added for *Lysinibacillus*. After room temperature gelation, incubation was at 30 °C for the time indicated.

#### Electron microscopy

A 6–9 mm segment of agarose gel-supported bacterial lawn was excised at the time indicated. The lawn was added to 0.5 ml of 4% formaldehyde, 1% glutaraldehyde, 0.11 M sodium phosphate, pH 7.3. After 2 h at room temperature, the gel was (1) washed for 5 min in 1.5 ml of 0.1 M sodium phosphate, pH 7.3 and (2) post-fixed for 30 min, at room temperature, in 1% osmium tetroxide in Zetterqvist’s buffer [16].

The specimen was then dehydrated in 1.5 ml of the following (in parentheses are number of changes/time [min] each): 70% ethanol (1/10), 95% ethanol (1/10), 100% ethanol (2/10) and 100% propylene oxide (2/10). Next, Epon 812, from Polysciences, was vacuum-infiltrated at room temperature in (1) a 50:50 mixture of resin with propylene oxide for 30 min, followed by (2) undiluted resin for 30 min. Finally, the resin was polymerized at 85 °C overnight, in a flat-embedding BEEM capsule.

An embedded gel was thin-sectioned with a Leica EM UC6 microtome and diamond knife. Sections were adhered to a 150-mesh copper grid, after color-selection [17] for ~ 100 nm thickness. Sections were stained with 7% uranyl acetate for 30 s, followed by Reynold’s lead citrate [18] for 20 s both in a microwave oven (0.035 W cm^−3^).

EM was performed with a JEOL JEM-1400 electron microscope. Images were recorded with an AMT image capture engine Version: 7. Boxed regions in figures were contrast enhanced.

### Results

#### Distribution of cells

**Fig. 1:**
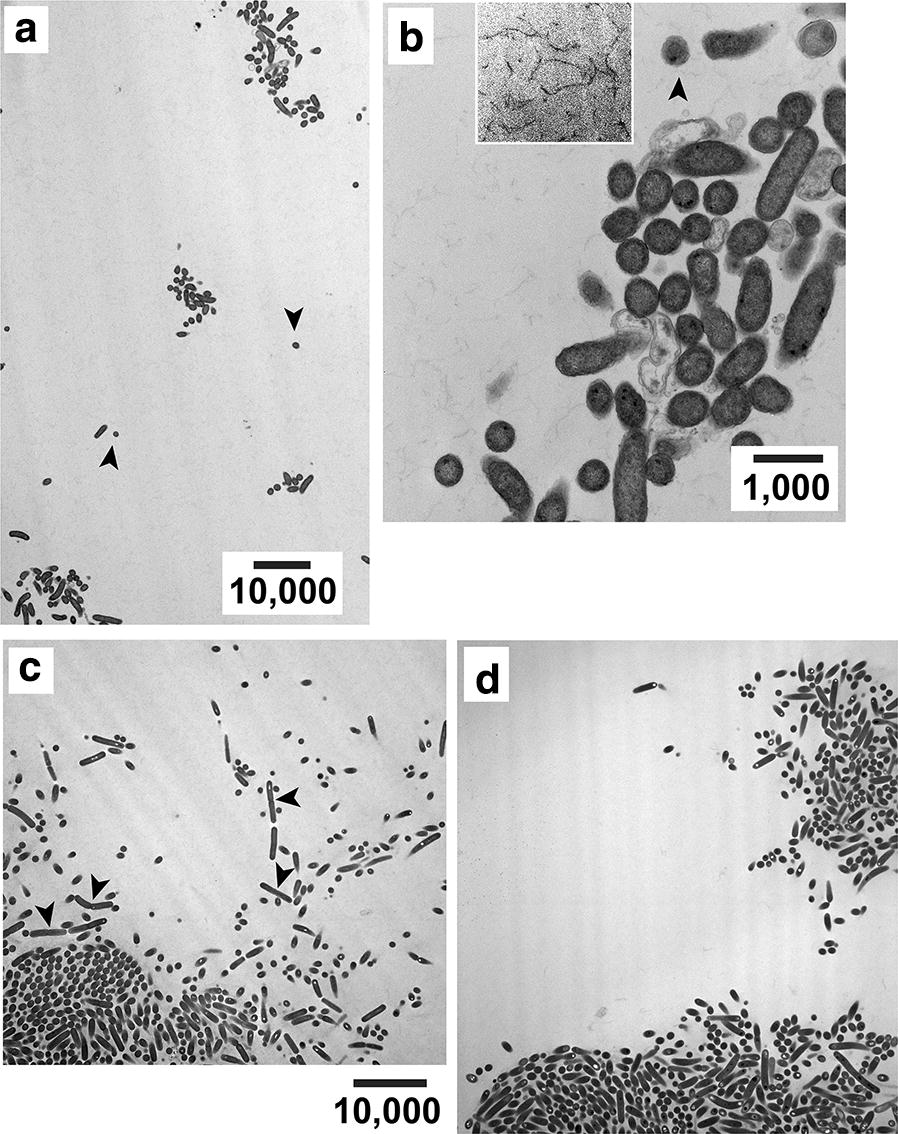
Bacterial clusters after in-gel propagation. EM of a thin section is shown after incubation in a 0.6% agarose gel of **a** *E. coli* for 16 h, **b** *Lysinibacillus* for 16 h, **c** *E. coli* for 72 h and **d** *Lysinibacillus* for 32 h. Magnification bar lengths are in nm

EM revealed that most cells (82% of 638 randomly selected) of a 16 h, 30 °C-incubated *E. coli* lawn were clustered (Fig. 1a). Clusters had 8–80 bacteria (average, 30, in 19 randomly selected clusters) with surrounding, inter-cluster, bacteria-free regions larger than clusters. The latter regions always had fibers indistinguishable from those previously seen [19] in agarose gels. These fibers (1) were best seen in higher-magnification micrographs (Fig. 1b; boxed region) and (2) indicated that gel breakage was not the source of the cell-free space.

The clusters were, in some locations, surrounded by individual cells (arrowheads in Fig. 1a, b). The latter could have resulted from (1) presence in a cluster that included cells not in the section, (2) post-replication migration away from a cluster and (3) non-replication.

Three characteristics of the clustering increased when the time of incubation was increased to 72 h: (1) percentage of cells cluster-associated (85–95%, depending on field), (2) number of cells per cluster (100–400) and (3) average compactness of the clusters. In Fig. 1c, a densely packed cluster at bottom left appears to be merging with a less densely packed cluster at bottom right. The result was indistinguishable for *Lysinibacillus* except that the larger clusters appeared earlier (32 h: Fig. 1d).

By EM, *Lysinibacillus* and *E. coli* cells were 550–730 nm wide and 2000–5000 nm long. This width is smaller than the 1000 nm obtained by light microscopy for *E. coli* in liquid culture [8], perhaps because of cell shrinkage during dehydration (“Methods” section).

#### Effects of clustered cells on gel fibers

**Fig. 2:**
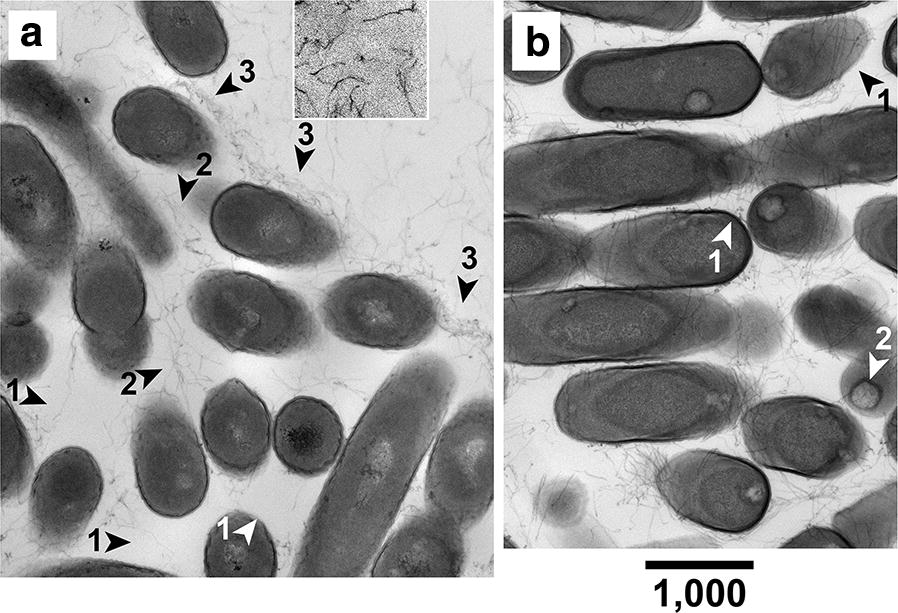
Dislocation of gel fibers. Dislocation of agarose fibers is observed in thin sections of a cluster of **a** *E. coli* grown for 72 h in a 0.6% agarose gel and **b** *Lysinibacillus* grown for 32 h in a 0.8% agarose gel (spores were seen in *Lysinibacilli*; arrowheads #2). Magnification bar length is in nm

For 72-h *E. coli* clusters in a 0.6% agarose gel, the density of inter-cell fibers varied from near-zero (arrowheads #1 in Fig. 2a) to density (arrowheads #2 in Fig. 2a) higher than the fiber density just outside of the cluster. Thus, within a cluster, growing cells appear to have dislocated fibers.

More dramatic evidence of gel fiber dislocation was finding of agarose fibers in bundles at the periphery (only) of *E. coli* clusters in a 0.6% gel. Arrowheads #3 indicate these bundles in Fig. 2a. All observed fibers were assumed to be agarose because no fibers were seen emanating from most regions of cell surfaces. Thus, the assumption is that bundling was caused by gel compression caused by cluster growth caused, in turn, by growth of cells within the cluster. This compression implied reactive force on compression-generating cells (Newton’s Second Law).

**Fig. 3:**
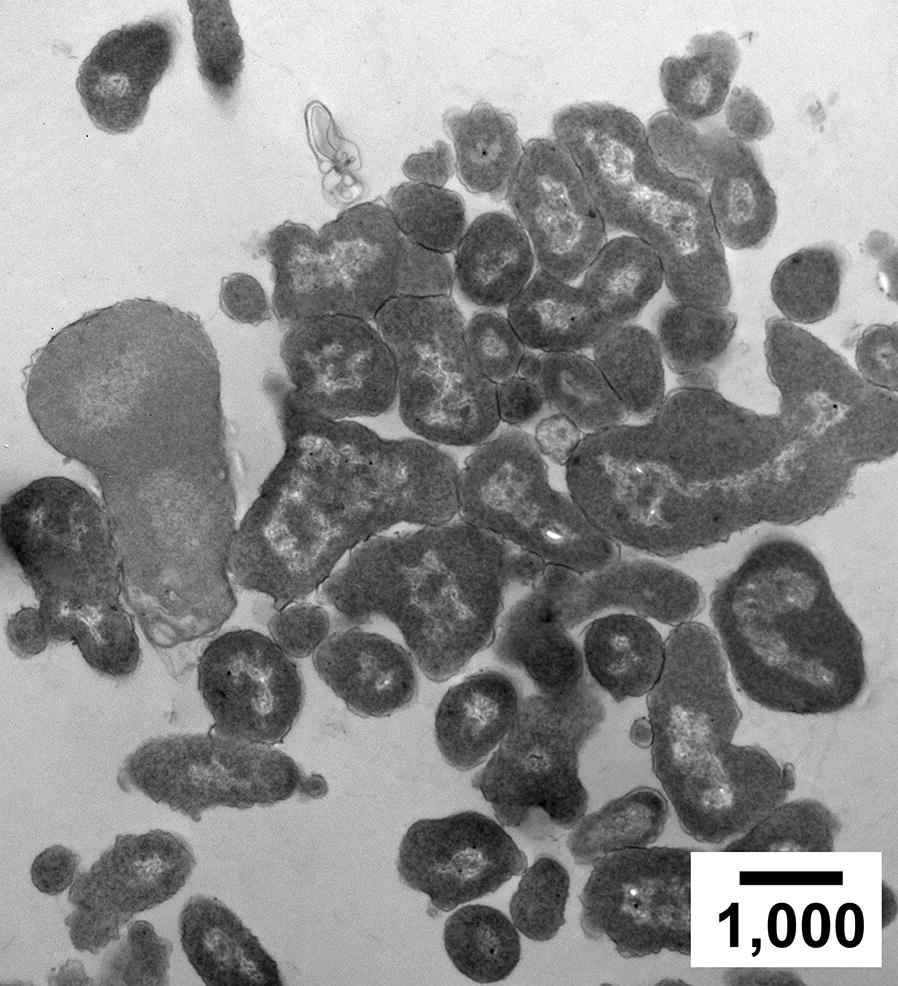
Effects of adding 0.94 M sucrose to the growth medium. Shape variability of cluster-associated *E. coli* cells is seen in a thin section of cells propagated for 48 h in a 0.6% agarose gel. Magnification bar length is in nm

Results with *Lysinibacillus* lawns in a 0.8% gel also led to the conclusion of fiber dislocation by growing cells. Comparable variability of fiber density was seen (Fig. 2b). Sometimes, both cell and relatively concentrated gel fibers were superimposed. Cell growth apparently caused these fibers to wrap around the cell (arrowheads #1 in Fig. 3b).

#### Some aspects of cell position and orientation within a cluster

Non-movement of cells after duplication in-gel should result in end-to-end chains of average length more than four cells. The reasons are that (1) progeny cells are arranged end-to-end immediately post-duplication [20] and (2) on average, more than two duplications occurred in the time of incubation. EM did sometimes reveal longitudinally sectioned chains two cells in length (Fig. 1c, arrowheads). But, longer chains were seen at a rate ~ 1% of the number of chains two-long. Thus, movement of cells usually occurred after duplication.

Additional evidence of movement was that the 32-h *Lysinibacillus* and the 72-h *E. coli* clusters often had a sub-cluster array of 10–50 neighboring cells transversely sectioned, as seen in Fig. 1c for *E. coli*. These arrays had to be generated by bacterial motion because propagation of inoculum-contained cellular aggregates did not cause them. Phase contrast microscopy revealed that aggregation was absent in the inoculum.

#### Sucrose in the growth medium: deviations of cell shape

We also performed EM of *E. coli* after in-gel propagation in medium with water activity (a_w_) lowered by inclusion of 0.94 M sucrose (a_w_ = 0.97 [21]). Some phage T3 mutants have displayed an informative phenotype in this medium [13]. Biofilms were sometimes favored by lowered a_w_ (cystic fibrosis-generating biofilms, for example [3]).

In this lowered-a_w_ medium, cell clustering was accompanied by cell shape polymorphism never observed in unsupplemented medium. After 48-h incubation, most cells lost their rod shape (Fig. 3). Others may have been shape-altered, but not sectioned in a plane that revealed altered shape. The shape appeared not to be pre-determined, but to be controlled, in part, by volume exclusion of neighboring cells.

### Discussion

#### In-gel distribution of cells of a lawn

The observed cell clustering must have explanation based on the surrounding gel fibers. The reason is that cluster-inhibiting competition for nutrients and oxygen increases as clusters become larger and more compact.

At least two possible gel-derived, cluster-encouraging effects are suggested by our observation that gel fiber dislocation accompanies bacterial growth (Fig. 2a, b). The first occurs via (1) variable local gel strength and (2) bacterial growth rate that increases as local gel strength decreases. The asymmetric shape of some clusters (Fig. 1a, b) is explained by (1) and (2), together with asymmetric zones of local gel weakness.

The second possible cluster-encouraging effect is bacterial growth-dependent force that (1) is exerted by clusters against gel fibers and (2) increases as cluster size and density increase. This force would promote additional cluster-associated bacterial growth.

A clustering-amplified force might occur via combining of forces generated by bacteria throughout the cluster and/or inhibition of growth at a cluster surface. In either case, clustered cells are force-biased to outgrow cells that are either un-clustered or in smaller clusters.

For completeness, we note the (untested) possibility that clustering is further promoted by cellular secretions. This possibility includes improved production and use of growth factors via specialization and cross-feeding.

The observed side-by-side clustering is impossible via cell duplication without further translational and rotational cell movement. The presumed driver is reactive force exerted by dislocated gel fibers on bacterial cells.

#### In-gel changes in cell shape at lowered water activity

The observation of sucrose-induced, neighboring cell-controlled shape distortion in Fig. 3 is, to our knowledge, the first such observation. We do not have evidence of mechanistic details. We hypothesize that neighboring cells control shape because the sucrose over-rides mechanisms for specifying rod-like shape, possibly by increasing intracellular pressure.

#### Consequences for understanding both in-gel phage propagation and biofilms

The clustering observed here renders certain that phages T3, T7 and G propagate in conditions that vary, when in laboratory-generated, agarose (and presumably agar) gel-supported bacterial lawns. Nutrient concentrations will vary with position in and near clusters. In addition, cells will experience a variable gel fiber-derived pressure.

Previous studies have considered the following biofilm-generating factors: (1) characteristics of bacterial attachment surfaces [22], (2) availability and type of nutrients [23], (3) availability of oxygen [24], (4) water flow [25], (5) electrical activity of cells [26] and (6) quorum-sensing of cells [22]. The data presented here implicate clustering not, to our knowledge, a factor previously considered. Clustering would cause biofilm-associated bacterial strains to be mixed primarily as clusters, not single cells.

### Limitations

1. We have not repeated the analysis performed here with biofilms.
2. Although the fibers in our images do not appear to emanate from bacterial cells and look like agarose fibers, a chance exists that some of them are secreted by the cells.
3. The levels of cluster-associated nutrients and oxygen have not been measured.

## Abbreviations

a_w_: water activity
EM: electron microscopy
2 × *P*_E_: diameter of the effective pore of an agarose gel
